# Does age at menarche influence final height in female adolescents?

**DOI:** 10.6026/973206300213363

**Published:** 2025-09-30

**Authors:** Arrthy S., Pushpa G., Pravallika P.

**Affiliations:** 1 Department of Physiology, Shridevi institute of medical science and research hospital, Tumkur, Karnataka, India; 2 Akash institute of medical sciences and research hospital, Devanahalli, Karnataka India

**Keywords:** Growth, puberty, short stature, adult height, adolescence obesity, BMI, parents height

## Abstract

Age at Menarche has shown decreasing trends worldwide in recent decades. This decline may potentially decrease target height and cause health related complications. A Cross sectional study was conducted among 150 female students aged above 18 years and background information was collected using a structured Questionnaire. Weight and height were determined using standard methods. It was observed that height in girls is influenced by age at menarche. Early menarche increases the risk of obesity among Indian girls.

## Background:

Growth is a complex biological process that involves both visible changes, such as an increase in height and body mass and internal developments like organ maturation [1]. These changes occur due to cellular activity influenced by genetic, environmental, and nutritional factors [2]. Skeletal growth progresses continuously from infancy through adolescence, following a characteristic pattern with two key growth spurts-one in early childhood and another during puberty [3]. Puberty is a critical phase of human development, marking the transition from childhood to adulthood [4]. In females, this process unfolds in four main stages: thelarche (breast development), pubarche (appearance of pubic hair), the pubertal growth spurt and menarche (first onset of menstruation) [5]. Menarche is considered a significant milestone of female puberty and is influenced by multiple factors, including genetics, ethnicity, geographical location, socioeconomic status and nutrition [6]. Over the past few decades, the average age of menarche has declined, particularly in Asian populations, largely due to improvements in nutrition [7]. Studies indicate that the mean age of menarche has decreased from 15-17 years in the 1960s to approximately 14 years in the 1980s, 12.6 years in the 1990s, and in some cases, as early as 10 years in recent times [8]. A study conducted among European birth cohorts reported a secular trend toward earlier menarche over time, with findings indicating that women who experienced menarche at a younger age attained a significantly shorter adult height compared to those with later menarche onset [9]. During puberty, the hormone estrogen plays a vital role in skeletal maturation, stimulating the fusion of growth plates (epiphyseal closure). This process typically occurs 2-3 years after menarche, leading to a significant reduction in height velocity [10]. As a result, girls who experience early menarche may also undergo earlier skeletal maturation, which could lead to a shorter final adult height [11]. Although growth is influenced by a variety of factors-including genetics, birth weight, nutrition, hormones, environment, and sleep and physical activity-height is primarily genetically determined [12]. Children grow toward a final height that is within a range called "the target range" [13]. The midpoint of this target range is called "the target height." The target height for females is calculated using the mid-parental height formula, which is subtracting 6.5 cm from the average of parents' heights [14]. While weight is largely influenced by environmental factors, height is predominantly inherited [15]. Adult height is also linked to overall health, with studies suggesting associations between stature and cardiovascular as well as pulmonary health [16]. Though numerous studies have explored the relationship between age at menarche (AAM) and growth, relatively few have focused specifically on target height as a reference parameter. Given that final adult height is predominantly influenced by genetic factors, it is reasonable to consider target height as a more accurate benchmark for assessing growth outcomes. Therefore, it is of interest to examine whether variations in AAM are associated with deviations from genetically determined target height in the Indian female population.

## Objectives:

[1] To assess the association between age at menarche and height difference.

[2] To assess the relationship between age at menarche and adolescene obesity.

[3] To determine the impact of lifestyle changes on age at menarche.

## Materials and Methods:

## Study design and participants:

A cross-sectional study was conducted using simple random sampling method in our institution between June-august 2024. Data was collected from 150 female students preferably localities enrolled in various medical courses (MBBS, BPT, Nursing, Paramedical).

## Inclusion and exclusion criteria:

## Inclusion criteria:

[1] Female student aged 18 years and above.

[2] Willing to participate with written informed consent.

## Exclusive criteria:

[1] History of chronic illness, endocrine disorders, congenital disorders, heart diseases.

[2] Intrauterine infection and intrauterine growth retardation.

## Data collection and measurements:

Data on the age at menarche (in years and month), involvement in sports from childhood, food habits (regarding high calorie food intake) in childhood and place of residence (rural/urban) were collected. Parents were invited to the college to provide details regarding their child's past illnesses, intrauterine growth retardation, birth history, parent's sports involvement in childhood, family income. All this information was collected through a structured questionnaire. Height and weight are measured with reference to NIHR Southampton Biomedical Research Centre Procedure, participants were instructed to remove their shoe and asked to wear minimal clothing, stand still and spread evenly while standing on the medical balance and Weight was measured to the nearest 0.1 kg. Height was measured by using a wall mounted stadiometer after checking its calibration. Participants were asked to remove shoes, hats, bulky clothing such as coats and sweaters, Undo hair styles and remove hair accessories that could interfere with the measurement. They were positioned with heels together, legs straight, arms at their sides and shoulders relaxed ensuring that the head, shoulders, buttocks and heels touching vertical surface [2]. Eyes should be looking straight ahead, chin not tucked or stretched too far back, the headpiece was brought down to the crown of the head with enough pressure to compress the hair and the measurement was taken with the measurers eyes parallel to the headpiece. Height was measured to the nearest 0.1 cm. The same protocol was followed to measure the height and weight of the participant's parents. As we mentioned earlier the target height for females is calculated using the mid-parental height formula, by subtracting 6.5 cm from average of parent's height [15].

Target height = Fathers height + Mothers height/2-6.5cms

## Statistical analysis:

The Collected data were entered into an excel sheet and analysed by using SPSS version 26. Participants were grouped based on AAM in to early (<12yrs), average (12-14 yrs) and late (>14 yrs) menarche categories. The height difference was calculated as the difference between the observed final height and target height. Descriptive statistics, including mean, standard deviation (SD) and sample size (n) were calculated for height difference within each menarche group. For girls who attained early menarche whose height difference is less than 0 one tailed t tests were used to find the significance. For average and late menarche groups two tailed t test was used to find the significance. AAM and BMI were treated as continuous variables. Pearson's correlation coefficient was used to evaluate the linear relationship between BMI and AAM. A Scatter plot was made to visually examine the relationship between two continuous variables and linear line to show the strength and direction of the relationship. Regression analysis was used to assess the association between two groups or variables (numerical data). A p<0.05 were considered as statistically significant. Spearman's rank correlation was applied to compare the AAM with categorical variable like sports activity, sleep time, screen time, junk food consumption and exercise.

## Results and analysis:

The difference between actual height and target height (height difference) was compared among girls with early, average and late menarche. It was observed that girls with early menarche had a mean height lower than their target height, but this difference was not statistically significant (p=0.10). The girls with average menarche had a mean height greater than their target height and this difference was statistically significant (p=0.03). Girls with late menarche also had a mean height greater than their target height, but the difference was not statistically significant (p=0.13) (Table 1 - see PDF, Figure 1 - see PDF). A Pearson's correlation analysis revealed a significant weak negative correlation between age at menarche and BMI (r=-0.246, p=0.001) suggesting a potential association between early menarche and increased risk of obesity in later life (Table 2 - see PDF, Figure 2 - see PDF). A spearman's rank correlation analysis revealed a significant weak negative correlation between AAM and screen time (Table 3 - see PDF). Though weak positive correlation was observed between AAM with exercise, sports activity, sleep time and a weak negative correlation was observed between AAM and intake of junk food, it was not statistically significant.

## Discussion:

In our study, it was found that height of girls who attained menarche at and above 12 years grow taller than the predicted height. These results suggest that timing of menarche may influence final height outcomes, with average menarche associated with greater deviation above the target height. In an epidemiologic study conducted by kang *et al.* concluded that final height in girls is influenced by age of menarche and early menarche increased the risk for adult short stature in young Korean females [17]. The underlying mechanism can be attributed to the early closure of the epiphysial growth plates due to the influence of ovarian hormone oestrogen, which is secreted during early puberty [18]. In contrast, delayed menarche allows continued growth in long bones, resulting in increased adult height. Though AAM is determined by both genetic and environmental factors, the secular trend in AAM suggests that the environmental factors are the major contributor of early menarche [19]. Like many other countries, India is also facing rapid decrease in the age of menarche and these changes might be due to improvement in nutrition and changes in socioeconomic conditions [20]. Dietary transitions, including a decrease in intake of coarse cereals, pulses, fruits and vegetables, an increase in intake of meat products and salt, coupled with declining levels of physical activity due to rapid urbanization have resulted in escalating levels of obesity [21] and many studies reports that girls with increase in BMI and more body fat reach menarche earlier [18, 22 and 23]. The "critical weight hypothesis" proposed by Frisch-Revelle *et al.* suggests that menarche occurs once a girl attains a specific body fat percentage (22%) or body weight (approximately 48kg) [24]. Leptin a hormone secreted by adipose tissue plays a crucial role in relaying information about fat mass distribution to the hypothalamus during puberty [25]. This in turn stimulates the pulsatile release of gonadotropin-releasing hormone (GnRH) in the hypothalamus, which serves as a signal for menarche [26]. Additionally, Gemelli *et al.* reported that menarche is strongly associated with changes in body composition, particularly increased body fat and muscle mass. These findings suggest that obesity is a significant contributing factor to early menarche [27]. In our study we have found that more screen time is associated with early menarche. Similar results were observed in a study by Li *et al.* which found that girls who spent more than seven hours per week on electronic devices were three times more likely to experience early pubertal development (EPD) [28]. Although the exact mechanisms remain uncertain in humans, animal studies suggest that sleep disruption due to screen exposure may lead to weight gain, which in turn contributes to early puberty. Additionally, exposure to artificial light can suppress melatonin secretion, thereby accelerating the release of pituitary gonadotropins, which are typically inhibited by melatonin, leading to early pubertal onset [29].

Interestingly, we did not find any significant association between menarcheal age and factors such as physical activity, exercise, sleep duration, soy intake, or junk food consumption. However, some studies indicate that diet quality can influence the timing of puberty. Carwile *et al.* found that high fructose consumption, particularly from sugar-sweetened beverages, was associated with earlier menarche among U.S. girls [30]. Berky *et al.* reported that a high animal-to-vegetable protein intake ratio during early childhood (aged 3-5) was linked to earlier menarche, even after controlling the BMI [31]. Additionally, fruit juices, which lack dietary fiber, may contribute to earlier menarche by reducing estrogen deconjugation in the gut and increasing its reabsorption [32]. Early-life feeding practices also play a role, with formula feeding being associated with a higher risk of early menarche, whereas breastfeeding appears to have a protective effect [33, 34]. Environmental factors, including exposure to endocrine-disrupting chemicals such as bisphenol A (BPA), polychlorinated biphenyls (PCBs) and phthalates, have also been implicated in early menarche and obesity [35, 36]. Although our study did not establish a link between exercise and menarcheal age, previous research has shown that regular physical activity (≥2 hours per day) can delay menarche by an average of one year in Columbian university women [37]. Furthermore, we observed that girls with early menarche had higher BMIs, reinforcing the strong association between early menarche and future obesity risk. A study by Sol Kang *et al.* reported a higher prevalence of obesity in girls with early menarche, with the likelihood of obesity being 1.85 times greater among those who attained menarche before age 12 [17]. This link is attributed to elevated plasma estradiol levels and reduced sex hormone-binding globulin (SHBG) levels during puberty, which promote adiposity [38].

The Bogalusa Heart Study further established that early menarche is associated with increased body fat and a heightened risk of metabolic syndrome (MetS) in young adulthood [39]. MetS is characterized by central obesity, insulin resistance, hypertension and dyslipidemia [40]. The primary mechanism linking early menarche to MetS is obesity, which leads to insulin resistance and an elevated risk of type 2 diabetes mellitus (T2DM) [41, 42]. Hyperinsulinemia, a consequence of insulin resistance, further predisposes individuals to cardiovascular diseases [43, 44]. Epidemiological studies suggest that non-alcoholic fatty liver disease (NAFLD) often precedes the onset of Met S and significantly increases morbidity and mortality due to cardiovascular disease (CVD) [45, 46- 47]. Additionally, a meta-analysis of 117 epidemiological studies found that an earlier onset of menarche increases the risk of breast cancer by 5% per year of earlier menarche [48]. Conversely, delaying menarche by two years has been associated with a 4% reduction in endometrial cancer risk [49]. Taken together, these findings indicate that childhood obesity plays a crucial role in triggering early puberty and menarche, driven by adipocyte-secreted hormones [50]. Moreover, obesity in childhood tends to persist into adulthood [51], increasing the risk of insulin resistance, T2DM, NAFLD, MetS, CVD and breast cancer [52]. Given these significant health implications, early menarche must be recognized as a public health concern that warrants appropriate intervention strategies [53, 54].

## Conclusion:

Age at menarche decides the final adult height of girls and early menarche is associated with increased risk of obesity in future is shown. Thus, we show that early menarche may lead to shorter stature and increased BMI which results in long-term health consequences. Lifestyle factor such as prolonged screen time is also linked to early menarche, while no significant associations were found with AAM and physical activity, sleep duration, soya intake, or junk food consumption.

## Implications:

Implementation of health education programmes in schools focussing on fiber rich diet and reduced high caloric food, Encouraging physical activity and regulating screen time might delay the onset of menarche. The BMI in children with early puberty has to be monitored to reduce health related complications.

## Limitations of the study:

Since it is a retrospective study, the cause of early menarche could not be made out clearly. So a prospective study in girls from childhood is recommended to know more about the factors affecting AAM.

## Ethical clearance:

Obtained from the institutional ethical committee.
